# NRas slows the rate at which a model lipid bilayer phase separates†
†Electronic supplementary information (ESI) available: See DOI: 10.1039/c3fd00131h



**DOI:** 10.1039/c3fd00131h

**Published:** 2014-06-06

**Authors:** Elizabeth Jefferys, Mark S. P. Sansom, Philip W. Fowler

**Affiliations:** a Department of Biochemistry , University of Oxford , South Parks Rd , Oxford , OX1 3QU , UK . Email: philip.fowler@bioch.ox.ac.uk ; Fax: +44 1865 613 201 ; Tel: +44 1865 613 200

## Abstract

The Ras family of small membrane-associated GTP-ases are important components in many different cell signalling cascades. They are thought to cluster on the cell membrane through association with cholesterol-rich nanodomains. This process remains poorly understood. Here we test the effect of adding multiple copies of NRas, one of the canonical Ras proteins, to a three-component lipid bilayer that rapidly undergoes spinodal decomposition (*i.e.* unmixing), thereby creating ordered and disordered phases. Coarse-grained molecular dynamics simulations of a large bilayer containing 6000 lipids, with and without protein, are compared. NRas preferentially localises to the interface between the domains and slows the rate at which the domains grow. We infer that this doubly-lipidated cell signalling protein is reducing the line tension between the ordered and disordered regions. This analysis is facilitated by our use of techniques borrowed from image-processing. The conclusions above are contingent upon several assumptions, including the use of a model lipid with doubly unsaturated tails and the limited structural data available for the C-terminus of NRas, which is where the lipid anchors are found.

## Introduction

The capacity of eukaryotic cells to sense signals and react is central to their ability to act in a cooperative way. Specific ligands are detected by a range of receptor proteins embedded in the cell membrane – these proteins then interact with other proteins to process, amplify and direct the signal to the appropriate location within the cell. Rather than being randomly distributed, these cell signalling proteins are localised to specific regions of the membrane and this is believed to be important for their function.^1^ In 1997, Simons and Ikonen^2^ proposed the “lipid-raft” hypothesis that identified small cholesterol-rich nanodomains as being the compartments into which cell signalling proteins partition. Although intuitively appealing, the lipid-raft hypothesis remains controversial,^3^ in no small part because rafts have not been directly detected. For example, although there have been many *in vitro* studies examining the ability of a lipid mixture to macroscopically phase separate into large ordered (L_o_) and disordered (L_d_) regions, this effect has never been directly observed *in vivo*. Little attention has been focussed on the other physical processes which also could be responsible for altering the local concentration of a protein: for example, the protein could interact with lipids, which could affect the dynamics of the bilayer. Also, the local curvature of a lipid bilayer could drive the preferential association of different proteins.

**Fig. 1:**
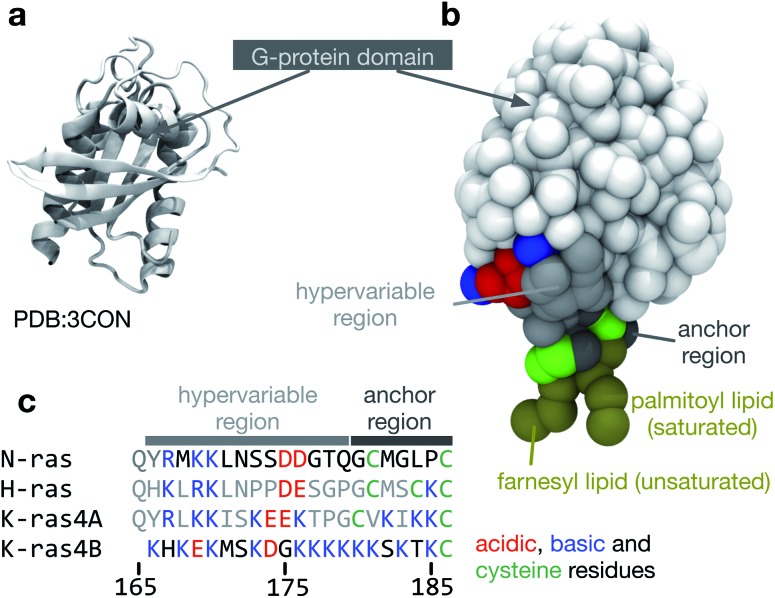
The structure of NRas. (a) The structure of the G-protein domain of NRas with guanosine diphosphate (GDP) bound^6^ at a resolution of 1.65 Å. Only residues 1 to 60 and 72 to 167 were resolved in the crystal structure. (b) The missing residues were modelled as described in the Methods and then converted to a coarse-grained MARTINI representation.^7^ A palmitoyl lipid was attached to Cys-181 and a farnesyl lipid was attached to Cys-186 at the C-terminus. (c) A multiple sequence alignment between the canonical members of the Ras family highlighting the cysteines (green), to which lipids can be attached and the large variation in acidic (red) and basic (blue) residues. The same colouring scheme is used in the other panels of this figure.

We shall focus here on NRas, a membrane-associated G-protein. Mutations of NRas, and the other members of the Ras family, are found to varying degrees in most human cancers,^4^ underscoring their centrality in many different cell signalling cascades and hence their physiological importance. NRas comprises a G-domain and a hyper variable region (HVR, Fig. 1) that mediates its interactions with lipid bilayers. In common with the other members of the Ras family, the C-terminal cysteine (Cys-186) is covalently and irreversibly attached to an unsaturated lipid (a farnesyl moiety).^5^ A second saturated lipid, usually a palmitoyl group, may be attached to Cys-181; this lipid modification can be reversed enzymatically. In this paper, we shall investigate using computer simulation how the protein itself affects the properties and behaviour of a phase-separating lipid bilayer and whether the segregation of NRas can be driven by the immiscibility of different lipid species.

Until recently, one could only simulate several hundred lipids for a few tens of nanoseconds, perhaps with a single protein, or protein complex (*e.g.* an ion channel), embedded, albeit with all atoms represented. With the advent of so-called ‘coarse-grained’ models for lipids and proteins and the continual growth in computational power, it has become possible in the past few years to simulate thousands of lipids for microseconds, a huge increase in capability in a short space of time. MARTINI is the most widely used coarse-grained forcefield^7^ and it is parameterised to reproduce a range of experiment results for the lipids (such as the area per lipid) and the amino acids (such as the partition free energy). MARTINI has been used to simulate the phase separation of mixtures of lipids and cholesterol^8^ and the behaviour of a range of proteins, including members of the Ras family, when inserted into phase-separating lipid mixtures.^9–11^

Unfortunately our ability to visualise and analyse such complex systems has not fully kept pace with these developments. Consider how one would determine if the lipid bilayer has separated into L_o_ and L_d_ domains and what their sizes are. Conventionally, spheres would be placed at the Cartesian coordinates of the different lipid atoms or coarse-grained beads, and an image rendered with a program such as VMD^12^ or PyMol. This will show if phase separation is complete – quantification is more difficult.

**Fig. 2:**
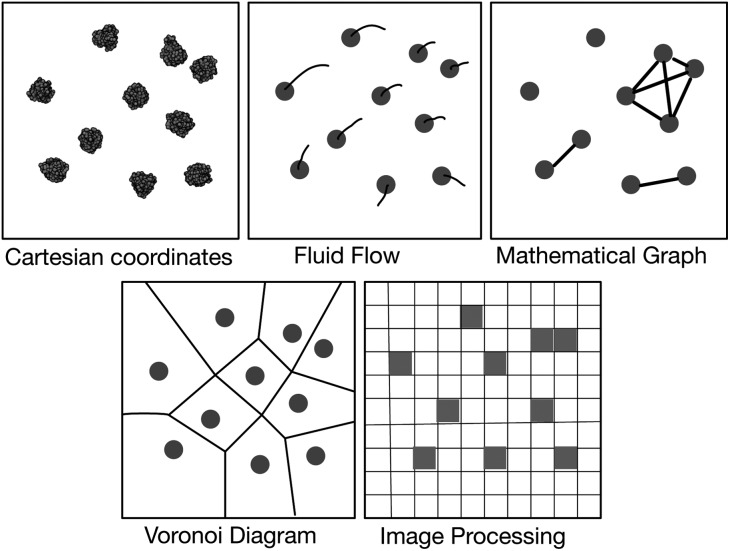
Schematic illustration of several paradigms for visualisation and analysis. The first image is a top view of ten NRas proteins taken from one of the simulations. Van der Waals spheres have been placed at the centres of mass of all the coarse-grained beads and an image rendered using VMD.^12^ All other images are illustrative.

Possible solutions come *via* the realisation that the vast majority of our existing visualisation and analysis tools are firmly rooted in the Cartesian paradigm and are not well suited to a study of this nature; alternative paradigms may be more appropriate (Fig. 2). The first of these borrows concepts from *fluid dynamics* and intuitively displays the velocity of particles using streamlines; this would help one study the diffusion and collective behaviour of lipids and, for example, if their motions correlate with embedded proteins. This is the subject of another paper at this Faraday Discussion meeting.^13^ The second is to construct a *mathematical graph* where, for example, the nodes are the centres of mass of the proteins and an edge indicates that two proteins are interacting. The largest connected cluster and other useful statistics can be easily retrieved from such a graph. Alternatively, one can represent the surfaces of the lipid bilayer as either a *Voronoi tessellation* or a *Delaunay triangular mesh* (the latter is the dual graph of the former and hence they are related). Voronoi tessellation has been extensively used to measure the area per each lipid in a simulation^14–17^ and has also been used to examine the phase separation of a mixed lipid bilayer.^18^ Finally, one may borrow ideas from the field of *image processing* and represent the bilayer as a two-dimensional array of pixels whose values, for example, could indicate the density of a particular molecular species. This last paradigm is the best suited to our current problem and, as we shall see, standard image processing techniques and libraries will allow us to easily detect where the edges between different phases lie.

## Methods

### Setup of phase-separating lipid bilayer

A previously equilibrated patch of 1500 coarse-grained POPC lipids^19^ was first tessellated to produce a square patch of 6000 lipids, 452 Å square. The sides of each patch fit perfectly together because the simulations are run with periodic boundary conditions. The lipids were then randomly ‘mutated’^19^ to achieve a 5 : 3 : 2 mixture of dipalmitoylphosphatidylcholine (DPPC, 751 lipids), dilinoleylphosphatidylcholine (DUPC, also known as DLiPC, 450 lipids) and cholesterol (299 lipids). A polyunsaturated lipid, such as DUPC, is required for phase separation using the MARTINI forcefield.^8,20^ This mixture has also previously been shown to spontaneously phase separate in a reasonable time.^8,10^ This patch was then solvated by the addition of 65 984 water beads, 2016 Na^+^ beads and 2016 Cl^–^ beads, yielding an ionic strength of ∼0.15 M and a total of 137 232 coarse-grained beads. An initial conformation was produced by minimising the energy of the system for 1000 steps using the steepest descents algorithm. Three 5 μs simulations of the lipid bilayer without any protein were run as controls using the GROMACS 4.6 molecular dynamics code^21^ and the MARTINI v2.1 coarse-grained forcefield.^7^

### Details of the molecular dynamics simulations

All non-bonded interactions were shifted to zero when the distance between beads was 12 Å. A timestep of 20 fs was used and the temperature was held at 310 K using a Berendsen thermostat with a time constant of 1 ps. The pressure was maintained at 1 bar by a Berendsen barostat, applied semi-isotropically, with a time constant of 1 ps and a compressibility of 3 × 10^–4^ bar.^22^ Coordinates of all molecules were written to disc every 2 ns.

### Construction of dually-lipidated NRas

As shown in Fig. 1, the available crystal structure of NRas^6^ is incomplete. The missing surface-exposed loop (residues 60 to 72) was modelled using FREAD.^23^ An initial structure of the hyper variable region (residues 167 to 179) was generated using Chimera^24^ and attached to the G-domain. The resulting nearly-complete atomistic structure was converted to a coarse-grained MARTINI representation^7^ and simulated for 0.1 μs in a 100 × 100 × 100 Å box containing 9565 water beads, 96 Na^+^ beads and 90 Cl^–^ beads. Finally, a configuration of the anchor region (residues 180 to 186) was generated in Chimera^24^ that satisfied a set of dihedral angles measured by solid state NMR of full-length NRas.^25^ A harmonic bond with an equilibrium length of 5 Å and force constant 10 kJ mol^–1^ Å^–2^ was also applied between the backbone beads of Cys181 and Leu184 to maintain the horseshoe-shape propensity that has been observed in numerous experimental studies.^25–27^ The resulting, unlipidated anchor was simulated in a 60 × 60 × 60 Å box containing 1113 water beads, 20 Na^+^ beads and 21 Cl^–^ beads to ensure the construct was stable.

All published studies that use the MARTINI coarse-grained forcefield to study how Ras proteins interact with lipid bilayers assume that farnesyl, a complex unsaturated lipid, can be represented as a linear sequence of 3 hydrophobic beads. We produced an improved set of MARTINI parameters for farnesyl, including the use of C4 beads to mimic the more polar nature of the double bonds, as described in the ESI.† This resulted in the coarse-grained beads being placed at the centre of mass of each isoprenyl unit, resulting in a naturally staggered geometry. Parameters and coordinates for palmitoyl were taken from the MARTINI parameters for DPPC.^28^

Farnesyl and palmitoyl were linked to Cys186 and Cys181 of the anchor *via* harmonic bonds of length 3.9 Å and force constant 50 kJ mol^–1^ Å^–2^.^10^ The equilibrium bond angles were 100 degrees with force constant 25 kJ mol^–1^ rad^–2^, conforming to the carbon–sulphur–carbon (CSC) bond angle in thioethers. In order to model carboxymethylation, the Cys186 backbone particle was converted from type Qa with charge –1 to Na (uncharged). Comparison of the membrane association of peptides with and without this change demonstrated it to increase the membrane insertion depth of the C-terminus (data not shown), consistent with experimental data.^26^ To assess the stability of the lipid-modified anchor, it was simulated alone in a 150-lipid DPPC bilayer for 100 ns. The dually lipidated anchor was attached to the C-terminus of the G-domain-linker construct using the usual parameters for backbone particle bonded interactions,^29^ and the full-length model simulated for 100 ns in a 600-lipid DPPC bilayer with 6226 water molecules, 179 Na^+^ and 174 Cl^–^ beads to assess stability of the construct and allow it to relax.

### Insertion of NRas proteins into lipid bilayer

Since the model membrane is symmetric, we shall insert copies of NRas into both leaflets, rather than just one leaflet as would be the case in a physiological, asymmetric membrane. Ten copies of farnesylated and palmitoylated NRas were positioned so their lipid tails were inserted into each leaflet of the initial conformation of the phase-separating lipid bilayer, making twenty copies in each simulation. The proteins were arranged in a hexagonal pattern to optimise the packing of the protein. Rather than delete the lipids whose beads where within van der Waals distances of the lipidated proteins, we instead gradually “phased-in” over 1000 timesteps all the lipidated NRas proteins using the free energy module of GROMACS. A soft-core van der Waals potential^30^ was used, with values of the soft-core parameter and power of 1.5 and 1.0, respectively. To provide room for the G-domains, a larger number of water beads (169 701) were added to increase the extent of the simulation unit cell along the *z* dimension, resulting in a total of 251 141 coarse-grained particles. Three 5 μs simulations were subsequently run of doubly-lipidated NRas. The simulation parameters are identical to those used for the control simulations of the lipid bilayers.

### Analysis using image-processing techniques

**Fig. 3:**
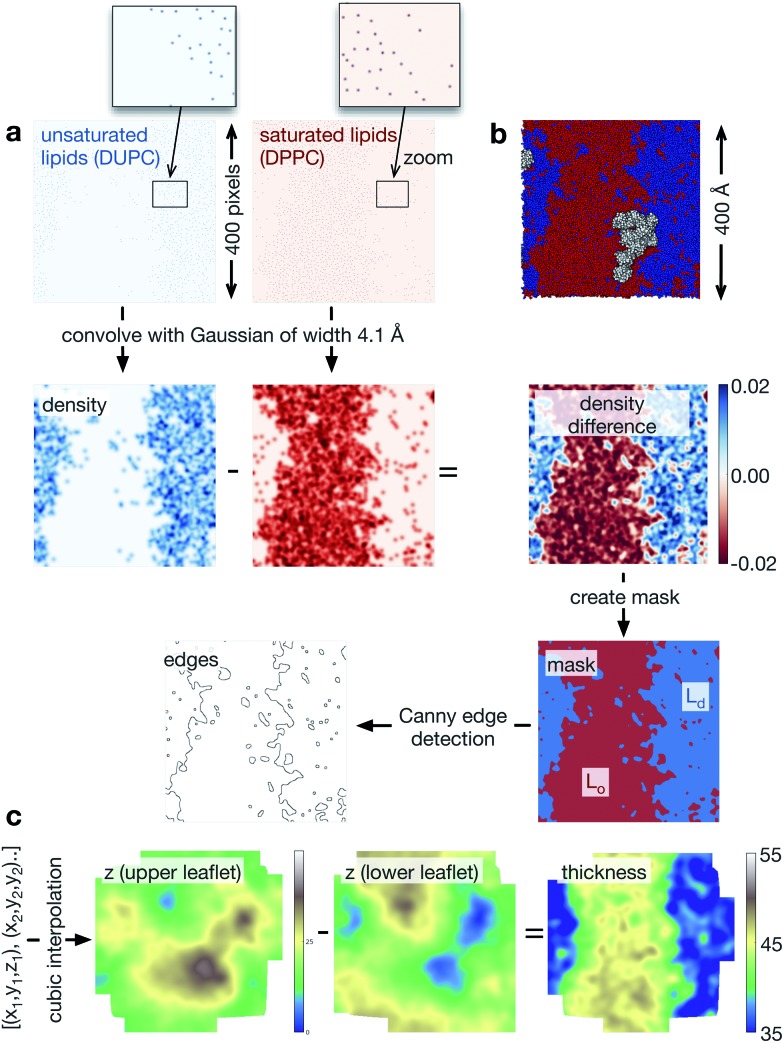
Workflow showing how the image processing techniques are applied to each lipid bilayer. (a) The sparse arrays showing where the phosphate beads of the unsaturated and saturated lipid species are convolved with a Gaussian of width 4.1 Å. The pixels in the initial sparse arrays are very small, so a region in each array has been magnified. The difference in these two arrays identifies qualitatively the L_o_ and L_d_ regions. If we identify any pixel with a value > mean density as belonging to the L_d_ phase and the remainder the L_o_ phase, then we generate a mask. Applying the Canny edge detection algorithm identifies the interface between the two phases. (b) For comparison, an image rendered by VMD of the same data is shown. (c) We can create the surface of each leaflet by interpolating between the coordinates of the lipid phosphate beads. The difference in these arrays naturally gives us the thickness of the bilayer. This is coloured using a topographical palette: blues and greens represent smaller values of the thickness, yellows and browns, larger values of the thickness. For simplicity, we have not shown the proteins, but a similar procedure can be applied to the coordinates of their centres of mass to create arrays of their ‘density’.

We used Python 2.6 for all of the analysis. The coordinates and trajectories were read into MDAnalysis,^31^ an open-source Python module that can read a wide range of coordinate and trajectory formats, including those produced by GROMACS. We also made extensive use of NumPy,^32^ a Python module that creates *N*-dimensional array objects, and SciPy,^33^ a library of functions that can act on NumPy arrays. Throughout, each pixel has dimensions 1 × 1 Å. First, two-dimensional NumPy arrays were created for each of the different species (DPPC, DUPC, cholesterol, protein) to cover the extent of the lipid bilayer. Since the initial configurations were all square and a semi-isotropic barostat was used, the lipid bilayers remained square, although their precise dimensions fluctuated during the simulations. Typically, the lipid bilayer measured ∼400 Å to a side, hence the NumPy arrays measured ∼400 × ∼400 pixels. A pixel was set to unity if the coordinates of either the PO_4_ bead (DPPC or DUPC), the ROH bead (cholesterol) or the centre of mass (protein) lay within its bounds (Fig. 3a). To give each species spatial extent (density), a Gaussian was then convolved with each NumPy array. We assumed that each lipid took up equal space in the leaflet and since there are 3000 lipids per leaflet and the bilayer is ∼400 Å square, this equates to a Gaussian 4.1 Å wide. When two NRas proteins interact, their centres of mass are ∼40 Å apart, hence, we chose the width of the Gaussian for the proteins to be 20 Å. To determine the extent of the L_o_ and L_d_ domains, we subtracted the density of the saturated lipid (DPPC) from that of the unsaturated lipid (DUPC, Fig. 3a). Next we created a mask which was unity everywhere this difference was greater than the mean, and zero everywhere else. This elegantly defined the L_o_ and L_d_ domains (Fig. 3a) and made subsequent analysis much more straightforward. Applying the Canny edge detection algorithm^34^ with a smoothing Gaussian of width 2.0 Å accurately identified the edges between the two phases (Fig. 3a).

Finally, to analyse the topology of the surfaces of the two leaflets of the lipid bilayer, we create a NumPy array of the height (*z*) of the phosphate beads (Fig. 3c). Since the pixel density is much higher than the lipid density, this is initially sparse, however, we used a cubic interpolation routine (from SciPy) to construct an array that describes the topology of the surface of either leaflet. The difference between the upper and lower surface naturally gave us the thickness of the bilayer.

## Results

### The lipid bilayer rapidly forms L_o_ and L_d_ domains

**Fig. 4:**
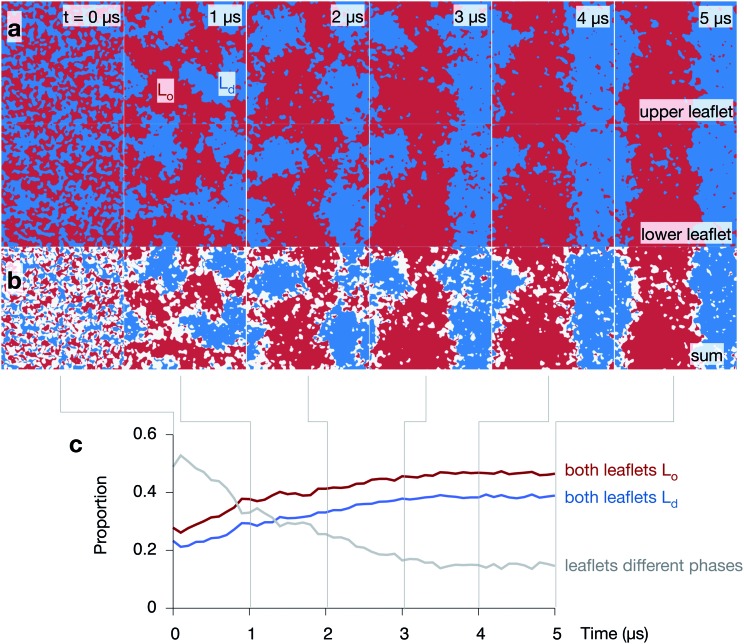
The 5 : 3 : 2 DPPC:DUPC:cholesterol mixture phase separates and the two leaflets are highly correlated. (a) Images showing how one of the control simulations undergoes spinodal decomposition (*i.e.* demixing) over the 5 μs. Both leaflets are shown and the ordered phase containing DPPC (and most of the cholesterol) is coloured red, whilst the disordered phase containing DUPC is coloured blue. (b) The sum of the two arrays shows the correlation between the two leaflets. Regions where both leaflets are ordered or disordered are coloured red and blue, respectively. Any region where the leaflets are in different phases are coloured white. The majority of the differences between the leaflets is found at the interfaces. (c) Initially the phase of around half of the bilayer does not match the phase of the other leaflet. This proportion falls as the bilayer undergoes spinodal decomposition, whilst the proportion of the bilayer where the phases on both sides of the bilayer match increases.

Let us first examine how the 5 : 3 : 2 DPPC:DUPC:cholesterol bilayer behaves by examining the three 5 μs control simulations of the lipid bilayer (Fig. 4 and Fig. S2 & S3 in the ESI†). During the course of each simulation, the regions of predominantly saturated (L_o_) and unsaturated (L_d_) lipids increase in size (Fig. 4a). After ∼3 μs, each leaflet has taken on a ‘striped’ appearance with (due to the periodic boundary conditions) one L_o_ stripe and one L_d_ stripe. The interface between the two phases remains very rough and there are small isolated islands of, for example, unsaturated lipids surrounded by saturated lipids. As one would expect, the cholesterol partitions primarily into the ordered L_o_ domain (Fig. S4 in the ESI†).

Adding the arrays of both leaflets shows that the formation of the L_o_ and L_d_ domains is highly correlated between the leaflets (Fig. 4b). The regions where there is a domain mismatch between the upper and lower leaflets occur mainly at or near to the interfaces between the two phases. The proportion of the bilayer where there is a domain mismatch is initially 49% but falls gradually as the L_o_ and L_d_ domains grow until a plateau of ∼15% appears to be reached after 4 μs (Fig. 4c) – we shall discuss this later. The other two control simulations behave in a similar manner (Fig. S2 & S3 in the ESI†).

### The L_o_ domain is thicker than the L_d_ domain

**Fig. 5:**
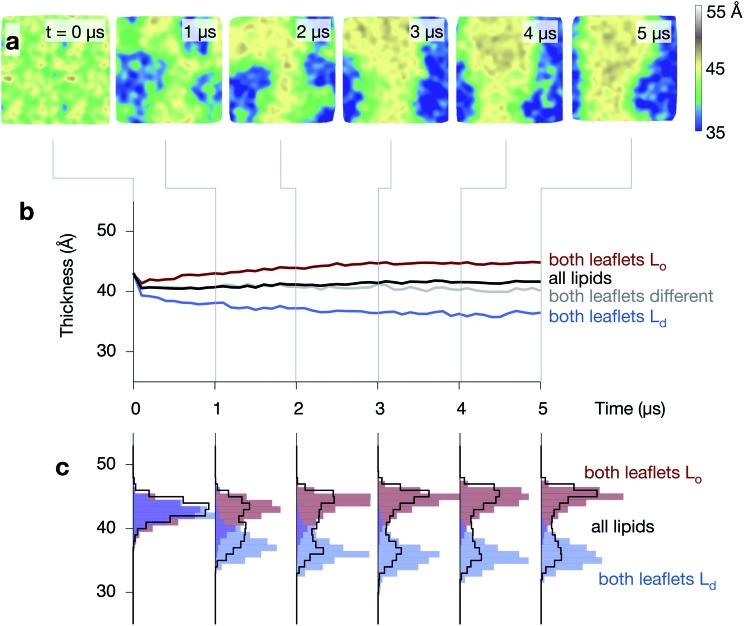
The ordered phase is thicker than the disordered phase. (a) A series of images of the thickness of one of the control simulations, showing how the central L_o_ strip is thicker than the surrounding disordered regions (compare to Fig. 4). (b) The average thickness, measured between the phosphate beads, (in black) remains unchanged at ∼41 Å during the formation of the L_o_ (red) and L_d_ (blue) domains, however, this hides the large difference in thickness between the two regions. (c) Examining the distributions shows how the complex behaviour develops.

The average thickness of the bilayer, measured between the phosphate beads, remains constant (∼41 Å) during the formation of the L_o_ and L_d_ domains, however, this hides significant differences between the thicknesses of the two phases that emerge during the simulations (Fig. 5 and Fig. S5 & S6 in the ESI†). The average thickness of the bilayer where both leaflets are disordered decreases, reaching ∼36 Å by the end of the simulation. At the same time, the average thickness where both leaflets are ordered increases, reaching ∼45 Å. Examining the distribution of bilayer thicknesses clearly shows the bimodal behaviour (Fig. 5c) and further suggests that the L_o_ and L_d_ domains get thicker or thinner, respectively, as the simulations progress and they get larger.

### NRas slows down the rate of spinodal decomposition

**Fig. 6:**
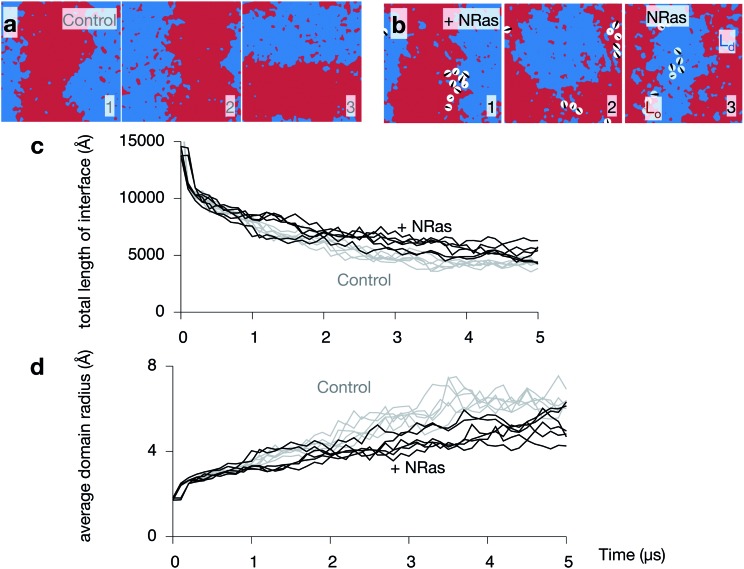
NRas slows down the spinodal decomposition. Images of the upper leaflet at 5 μs of the (a) three control and (b) three NRas simulations. One can also see how the banding occurs stochastically along either the *x* or *y* axes. (c) The total length of the interface between the L_o_ and L_d_ phases decreases during the simulations. Both leaflets for all simulations are plotted. NRas appears to retard this effect (grey lines). (d) Plotting the average radius of a domain more clearly shows this effect.

Since NRas has two lipid anchors, one saturated and one unsaturated, it is not clear what effect its presence in one of the two leaflets will have on the dynamics of the phase separation observed in purely lipid bilayers. Images taken from all three NRas simulations are shown in Fig. S7 in the ESI.† Comparing the upper leaflets of the three control simulations after 5 μs (Fig. 6a) to the upper leaflets of the three NRas simulations after 5 μs (Fig. 6b) is inconclusive; phase separation has occurred in both cases, although ‘stripes’ have not formed in the second NRas simulation, suggesting the process has been retarded. The total length of the interface between the L_d_ and L_o_ domains is a simple measure of the degree of phase separation. It rapidly decreases as the domains coarsen (Fig. 6c). However, when ten copies of NRas are added to each leaflet, we find that there are longer interfaces between the domains when NRas is present, compared to when it is not. A similar trend is observed when we plot the average radius of each domain against time (Fig. 6d); the average radius is reduced when NRas is added.

Whilst the proportion of the bilayer that has matched domains in both leaflets (Fig. 4c) appears to reach a plateau after 3–4 μs, the length of the interface and the average domain radius are still decreasing and increasing, respectively, after 5 μs. The theory of spinodal decomposition suggests that a system passes through several regimes, each of which follows a power law, *L*(*t*) ∼ *t* ^*α*^, where *L*(*t*) is the characteristic length and *α* is a parameter that varies between regimes. This disparity suggests that perhaps the consolidation and coupling of the domains in the two leaflets is one regime, but the overall demixing has not yet reached completion, *i.e.* reached equilibrium. If we examine logarithm plots of the total length of the interface and the average domain radius (Fig. S8 in the ESI†), we see that there is a small change in gradient around 2–3 μs, which is consistent with the above suggestion. The evidence is not compelling, however, and confirmation requires much longer, and possibly larger, simulations.

Since we cannot be sure that the phase separation is complete, we cannot rule out the possibility that the lipidated protein is altering the thermodynamics of the process. If true, then the equilibrium conformation of the bilayer would be different in the presence of protein. We can conclude, however, that NRas is, at the very least, retarding the rate at which the L_o_ and L_d_ domains grow.

### NRas localises to the interface between the L_o_ and L_d_ domains

**Fig. 7:**
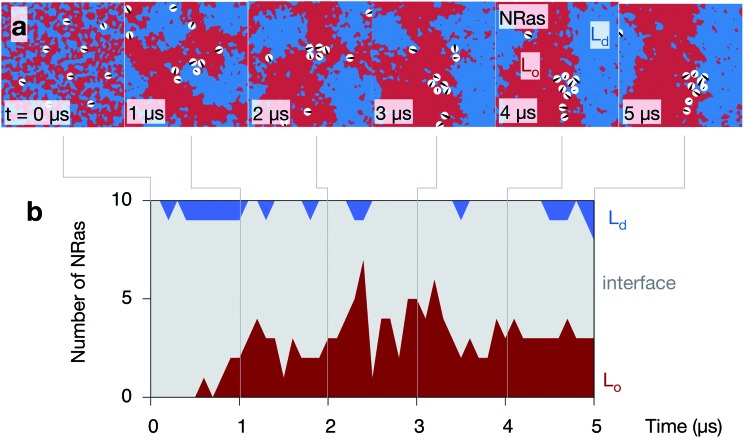
NRas localises to the interface between the L_o_ and L_d_ domains. (a) A series of images of the upper leaflet of one of the control simulations showing the localisation of the ten NRas protein (shown as white circles). (b) A protein is defined as belonging to one of the two phases if the 20 Å footprint of the protein contains >80% of one phase or the other, otherwise it is categorised as interfacial.

Inspection of the upper leaflet of one of the NRas simulations suggests that the lipidated protein prefers the interface between the two domains (Fig. 7a). Examining images of the other leaflet and the other simulations appears to confirm this (Fig. S7 in the ESI†). As mentioned in the Methods, the spatial extent of each protein is described by a Gaussian of width 20 Å. Examining the proportion of each domain under each of these Gaussians allows us to characterise whether each protein is sitting in the L_o_ domain (defined as >80% L_o_), the L_d_ domain (>80% L_d_) or at the interface (anything else). Since the lipids in the starting configuration are randomly mixed, 5/8th of the lipids under each protein are DPPC and hence all proteins are initially classified as interfacial (Fig. 7b). As the simulations progress and the domains coarsen, the number of proteins belonging to the L_o_ phase increases to ∼3 out of the 10 proteins. Very few proteins (0 or 1) are found in the L_d_ phase with the remainder (5 or 6), which is the majority, sitting at the interface between the L_o_ and L_d_ domains. With one exception, the majority of the proteins partition to the interface in the other leaflets of the other simulations (Fig. S9 in the ESI†).

## Discussion

Our simulations suggest that the cell-signalling protein NRas preferentially localises to the interface between L_d_ and L_o_ domains, which is perhaps not surprising since one of its two lipid anchors is saturated (palmitoyl), whilst the other is unsaturated (farnesyl). This is consistent with other simulation studies of NRas and other members of the Ras family that started from lipid bilayers that had already undergone phase separation.^10,11^ We infer that NRas is reducing the line tension between the two immiscible lipid species and that this effect is responsible for reducing the rate of spinodal decomposition when NRas is present – this is the first time this effect has been directly observed using simulation. The preference NRas appears to have for the interface between domains will automatically increase the clustering of the protein compared to if it were randomly distributed. There may be additional effects causing NRas to segregate yet further, but these are likely too subtle to be detected using this coarse-grained methodology.

A 5 : 3 : 2 mixture of DPPC:DUPC:cholesterol rapidly undergoes spinodal decomposition; domains form which rapidly coalesce, grow and coarsen. Since DPPC is saturated and DUPC is unsaturated, these represent ordered (L_o_) and disordered (L_d_) regions and we observe the former to be ∼9 Å thicker than the latter. Interestingly, there appears to be a correlation between the thickness (or thinness) of each domain and the extent of the phase separation *i.e.* the larger the domains, the thicker (L_o_) or thinner (L_d_) they become. This effect is not pronounced and requires further investigation. As expected, the majority of the cholesterol partitions into the ordered domain and therefore contributes to the disparity in thickness between the domains. We also observe a strong coupling between the two leaflets of the bilayer; domains in both leaflets tend to form in a synchronised manner such that, *e.g.*, L_o_ domains are found on top of L_o_ domains.

The introduction of coarse-grained approaches, combined with the continued increases in computational power, is permitting the study of ever larger patches of membrane. At 6000 lipids, this study is 3–4 times the size of previous studies.^10,11^ This is important as, since the simulation unit cell is periodic, the dimensions of the bilayer constrain the maximum size of the coarsening lipid domains. Simulating what is currently considered a large patch of membrane has hence enabled us to study the process of spinodal decomposition in more detail than we would have otherwise been able to. A study of comparable size has also been recently published examining the aggregation of HRas.^35^

We have borrowed techniques from image-processing to analyse our simulations. This has not only simplified the analysis of the simulations but also enabled us to answer questions that would have been, at best, very challenging had we tried to use conventional Cartesian-based methods. For example, estimating the length of the interface between the L_o_ and L_d_ domains relies on our use of the Canny edge detection algorithm.^34^ Once the edges have been found, however, measuring the length of the interface is a single line of Python code, since one just has to sum the elements in an array.

Central to our approach is the use of existing Python libraries and code, something that has been made possible by MDAnalysis,^31^ a Python module that reads most molecular simulation coordinate formats. Once one can retrieve and store in Python the Cartesian coordinates of the different molecular species from a simulation, one has access to the full range of well-documented, optimised and powerful Python modules. Here we have made extensive use of NumPy^32^ and SciPy^33^ and also scikit-image for image processing. Furthermore, the *N*-dimensional array object that NumPy creates dramatically simplifies the code since many complex operations become single lines. The use of existing, optimised libraries meant that the Python code that performed all the analysis and produced all the images in Fig. 4–7 only required ∼20 s per frame on a single CPU. Our approach is in contrast to the more traditional method of writing compiled standalone programs, which is time-consuming and more likely to introduce errors. These disadvantages will only grow as simulations and the questions we ask of them become ever more complex. Python is, of course, not the only possibility; MATLAB also has many similar libraries and tools.

We emphasise that all the visualisation and analysis paradigms listed in Fig. 2 are currently possible using Python. Mathematical graphs are straightforward to construct and interrogate using NetworkX,^36^ another Python module, whilst Voronoi tesselations and Delaunay triangular meshes can be built using SciPy.^33^

Despite its prevalence in the field, DUPC, which has unsaturated bonds in both aliphatic tails, is not a physiological lipid. It was adopted after it was shown to be required for phase separation to occur within microseconds.^8,20^ Unfortunately, this undermines the generality of the conclusions we draw, since it is possible that such rapid demixing as modelled here, does not occur *in vivo*. There is at present, however, no other way of modelling the formation of L_o_ and L_d_ phases using a coarse-grained approach that retains some chemical specificity of the lipids and the proteins. Our simulations therefore assume that this type of behaviour occurs in cells. The simulations, of course, could be longer, however 5 μs of a bilayer of 6000 lipids is already at the cutting-edge of what can be achieved, and it is preferable to have repeats of each simulation rather than a single, very long simulation. The use of a simulation box of finite size places an upper limit on the length of any features observed. This, when combined with the temporal resolution of the simulations, naturally places limits on the phenomena that can be modelled. We are, of course, assuming that all relevant physiological effects occur on the 10 nm length scale and 5 μs timescale.

As we have described, the available crystal structure of NRas is incomplete.^6^ Although we carefully constrained a model of the remainder of the protein using other available biophysical data, it is possible that our coarse-grained model of NRas is inaccurate. In particular, the relative separation of orientation of the two cysteine residues that are lipidated might be different. Fortunately, these two residues are only five amino acids apart so they must be in proximity to one another, regardless of the exact structure.

Our analysis also assumes that we can represent a lipid bilayer by a two-dimensional image, which is true only if the bilayer does not undulate significantly. Once this occurs, the link is broken between the Cartesian coordinates of the simulation unit cell and the local coordinate frames on the surface of the lipid bilayer, making analysis difficult. Fortunately, our bilayers do not significantly undulate, but this assumption limits the applicability of some of our methods. Finally, it is likely that there are subtle effects that are not described by the MARTINI coarse-grained forcefield. It is also possible that some of the effects we have observed are also artefacts, for example, more work on the observed clustering of NRas is required.

In future work, we will analyse in more detail the observed phase separation. The theory of spinodal decomposition posits that phase separation can be divided into several regimes, each of which conforms to a dynamic scaling hypothesis, *i.e.* its characteristic length, *L*(*t*), has a simple power law time dependence, *L*(*t*) ∼ *t* ^*α*^, where *α* is a parameter that varies between regimes. The curvature of the lipid bilayer has also been suggested to play a role in the segregation of Ras family proteins. Several methods to measure curvature have been recently introduced^17,18^ but these are at a nascent stage and require refining before they can be used.
