# Supplementary material for: NRas slows the rate at which a model lipid bilayer phase separates
Source: Faraday Discuss. 2014 Jun 6;169(1):209–23. doi: 10.1039/c3fd00131h (PMC4224576; doi:10.1039/c3fd00131h)
Supplement: Supplementary file 1 [file FD-169-C3FD00131H-s001.pdf]

Electronic Supplementary Material (ESI) for Faraday Discussions.
This journal is © The Royal Society of Chemistry 2014

NRas slows the rate at which a model lipid bilayer phase separates: Electronic Supplementary Information (ESI)

Elizabeth Jefferys, Mark S. P. Sansom and Philip W. Fowler*

Supplementary Methods - Parametrisation of the Farnesyl group

We first retrieved the lowest energy conformer of farnesyl methyl ether from the PubChem database (CID 5365855).[1] A library of 5 858 low-energy conformations was generated using Confab.[2] The centre of mass of the three isoprene units in each conformer was calculated and the angle θ between them measured (Fig. S1). The farnesyl beads were then positioned at the centres of mass of each isoprene unit in the lowest energy conformer to generate a starting farnesyl structure with a more realistic staggered conformation. The beads are joined via a harmonic bond of length 3.9 Å and force constant 50 kJ mol^{-1}Å^{-2} to the Cys186 side chain particle in the palmitoylated NRas anchor model. Eight copies of this dually lipidated construct (four per leaflet) were inserted into a symmetric bilayer comprised of 751 DPPC, 450 DUPC and 299 cholesterol and the system solvated, populated with Na^{+} and Cl^{-} ions to a total concentration of 0.15 M and subject to 5000 steps steepest descent energy minimisation.

A series of simulations were carried out using this test system in order to deduce appropriate parameters for the farnesyl group. In the Martini model for DUPC, the C4 particle type is used instead of C1 to mimic the more polar nature of double as compared to single bonds.[3] Following this line of thinking, both C3 and C4 beads were tested for use in the farnesyl model, in addition to a range of equilibrium bond angles

This test system was also employed to assess the elected method for modelling carboxymethylation; conversion of C-terminal backbone bead from Qa particle type with -1 charge to Na with no net charge. Two simulations were set up to test this; in one the C-

Department of Biochemistry, University of Oxford, South Parks Rd, Oxford, OX1 3QU, UK. Fax: +44 1865 613 201; Tel: +44 1865 613 200; E-mail: philip.fowler@bioch.ox.ac.uk

terminus was unmodified whereas in the other it was methylated using the afore mentioned conversion. The system was simulated for 1 μs, and the angle θ in the resulting simulations was measured using the Python-based MDAnalysis tools.[4] The distributions suggest that a bond angle of 125 degrees is optimal. Additionally, development of AT farnesyl parameters is an important next step, which would facilitate the examination of farnesylated proteins such as Ras in more detail.

References

1 E. E. Bolton, Y. Wang, P. A. Thiessen and S. H. Bryant, in *Annual Reports in Computational Chemistry*, American Chemical Society, Washington, 2008, vol. 4, ch. 12, pp. 217–241.

2 N. M. O'Boyle, T. Vandermeersch, C. J. Flynn, A. R. Maguire and G. R. Hutchison, *J Cheminformatics*, 2011, **3**, 8.

3 H. J. Risselada and S. J. Marrink, *Proc Natl Acad Sci U S A*, 2008, **105**, 17367–72.

4 N. Michaud-Agrawal, E. J. Denning, T. B. Woolf and O. Beckstein, *J Comput Chem*, 2011, **32**, 2319–2327.

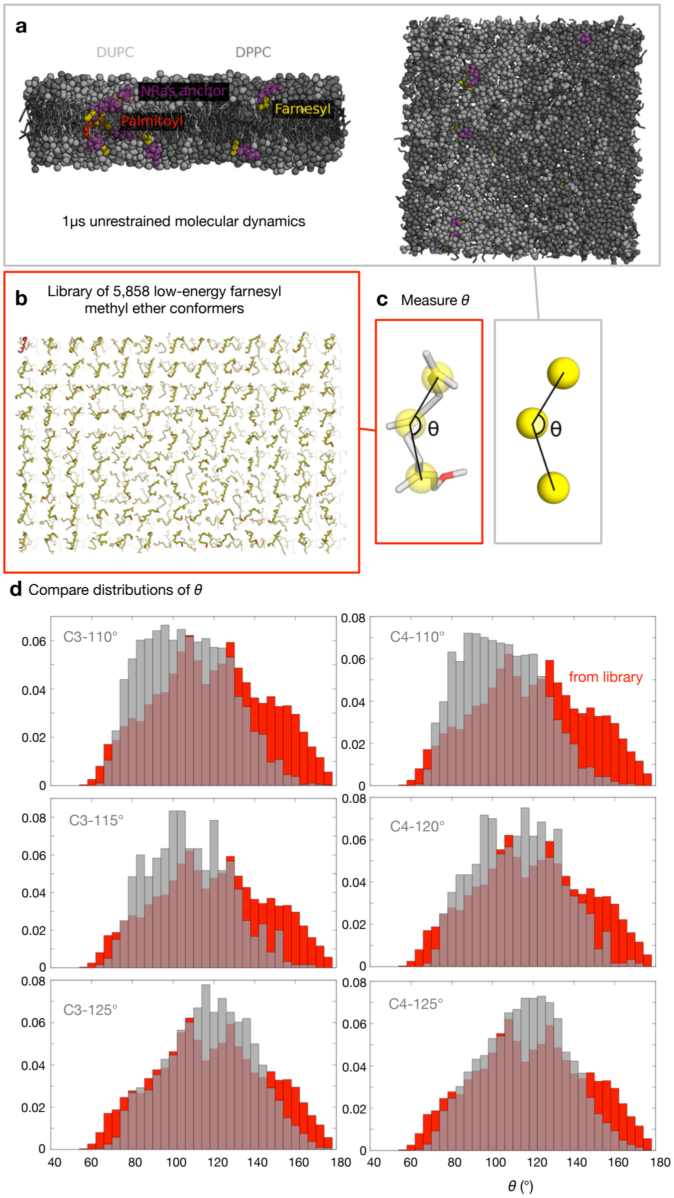

Fig. S1 Procedure for determining Workflow showing how the image processing techniques are applied to each lipid bilayer. (**a**) The sparse arrays showing where the phosphate beads of the unsaturated and saturated lipid species are convolved with a Gaussian of width 4.1 Å. The difference of these two arrays identifies qualitatively the L_o and L_d regions. If we identify any pixel with a value > 0 as belonging to the L_d phase and the remainder the L_o phase, then we generate a mask. Applying the Canny edge detection algorithm identifies the interface between the two phases. (**b**) For comparison an image rendered by VMD of the same data is shown. (**c**) We can create the surface of each leaflet by interpolating between the coordinates of the lipid phosphate beads. The difference of these arrays naturally gives us the thickness of the bilayer. For simplicity we have not shown the proteins, but a similar procedure can be applied to the coordinates of their centres of mass to create arrays of their 'density'.

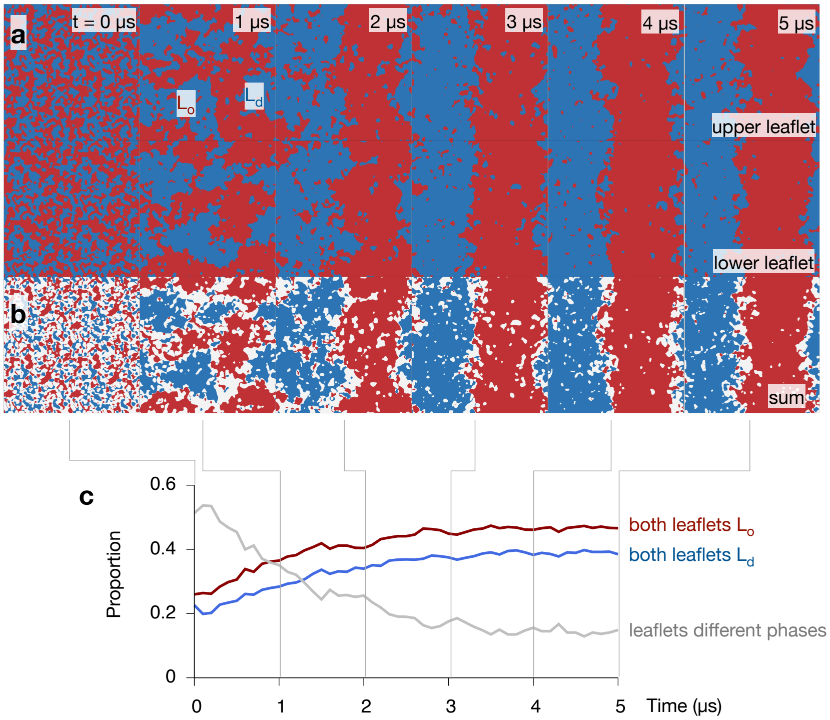

Fig. S2 The 5:3:2 DPPC:DUPC:cholesterol mixture in the second of the three control bilayer simulations also phase separates and the two leaflets are highly correlated. For the legend, see Fig. 4. in the main body of the manuscript

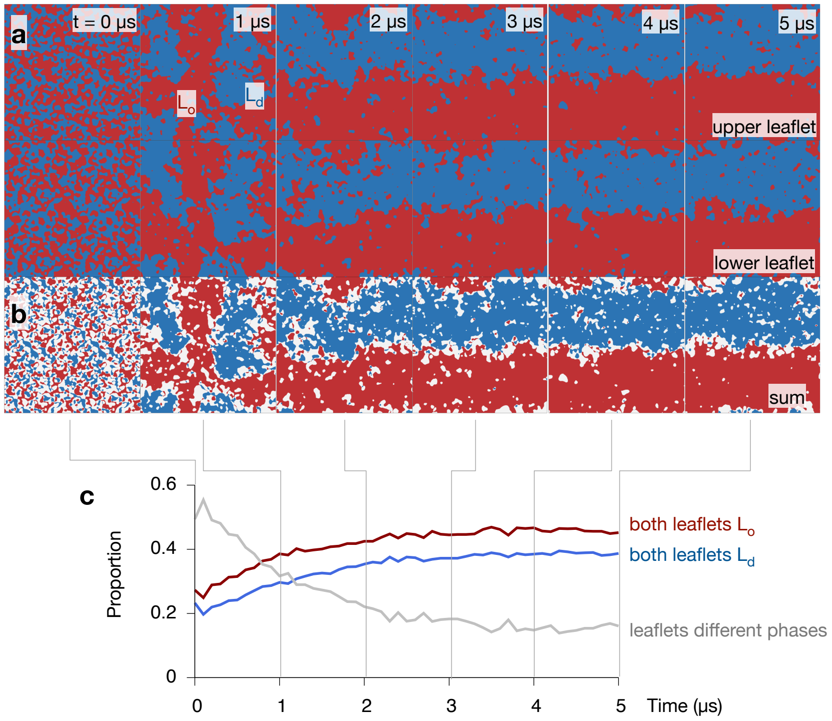

Fig. S3 The 5:3:2 DPPC:DUPC:cholesterol mixture in the third of the three control bilayer simulations also phase separates and the two leaflets are highly correlated. Note that the stripes have formed left-right, rather than top-down in this case. For the legend, see Fig. 4. in the main body of the manuscript

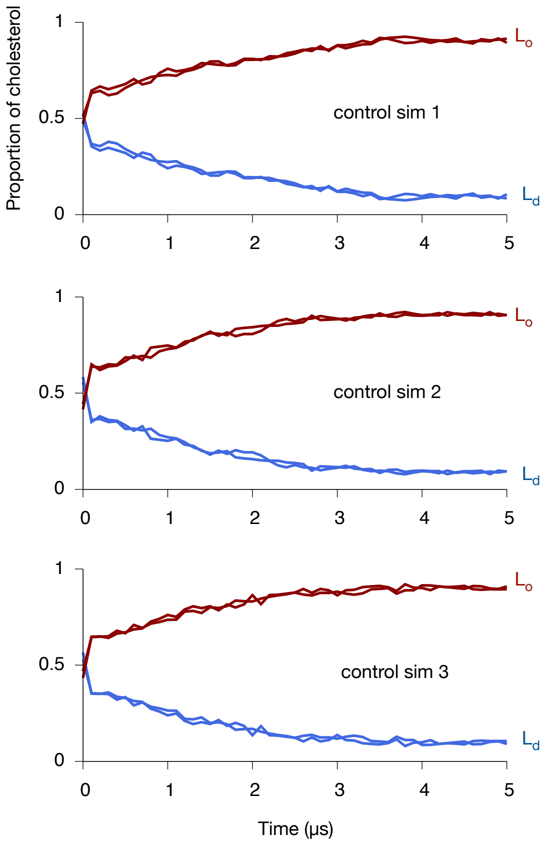

Fig. S4 Cholesterol partitions mainly into the L_o phase. The proportion of cholesterol that partitions into each phase is show for the three control lipid bilayer simulations. In all cases, $\sim$85 % of cholesterol ends up in the L_o domain after 5 μs.

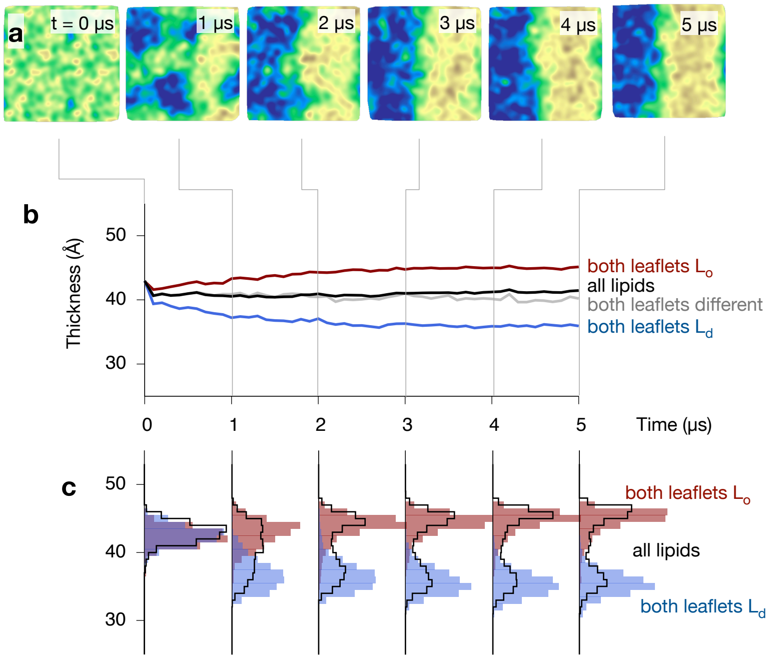

Fig. S5 The ordered phase is thicker than the disordered phase in the second of the three control bilayer simulations. For the legend, see Fig. 5. in the main body of the manuscript

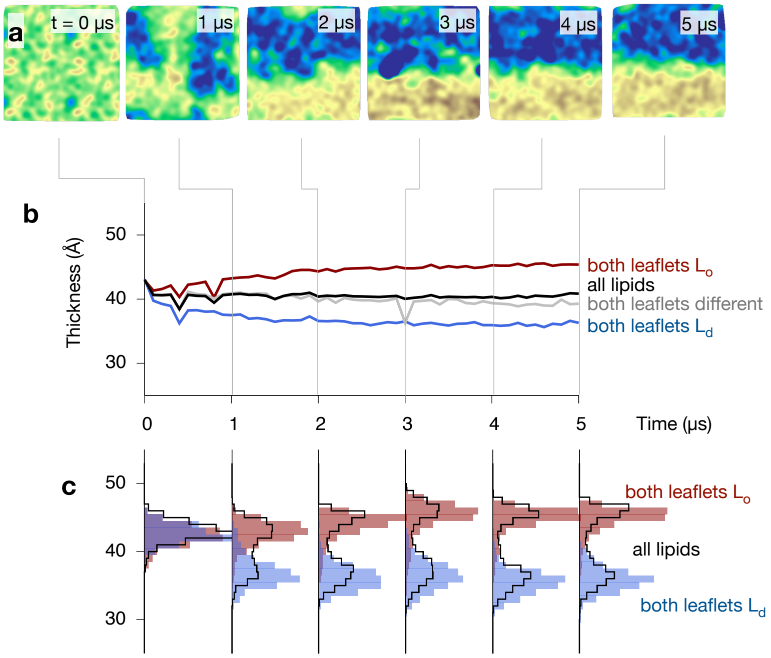

Fig. S6 The ordered phase is thicker than the disordered phase in the second of the three control bilayer simulations. For the legend, see Fig. 5. in the main body of the manuscript

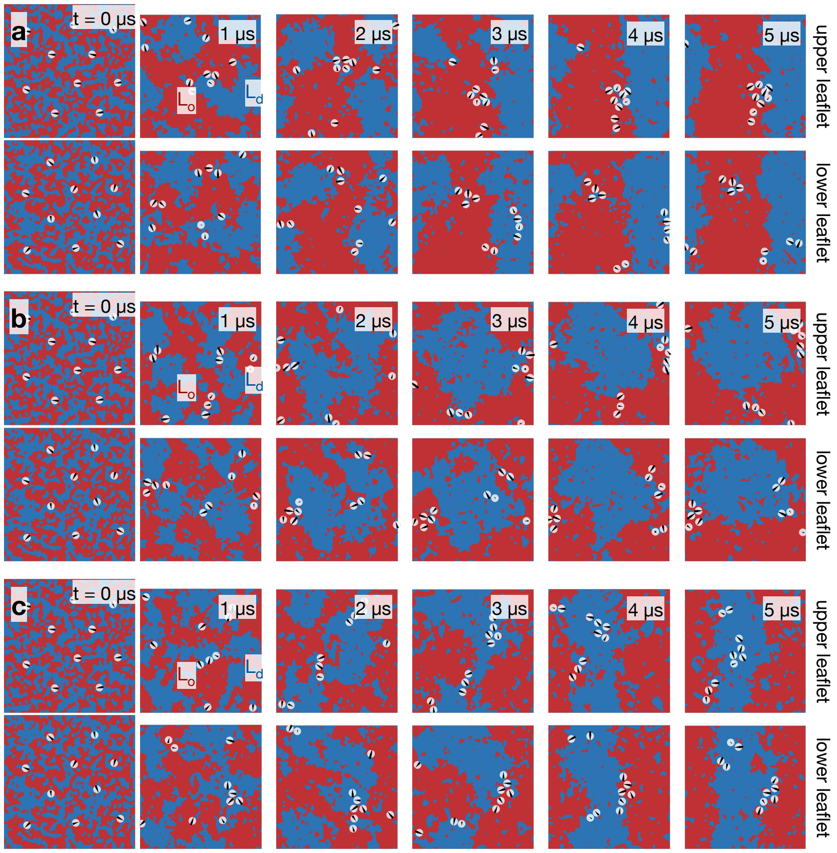

Fig. S7 Images from the three simulations with ten NRas proteins in each leaflet. The ordered and disordered domains are coloured red and blue, respectively, whilst the proteins are depicted as white circles.

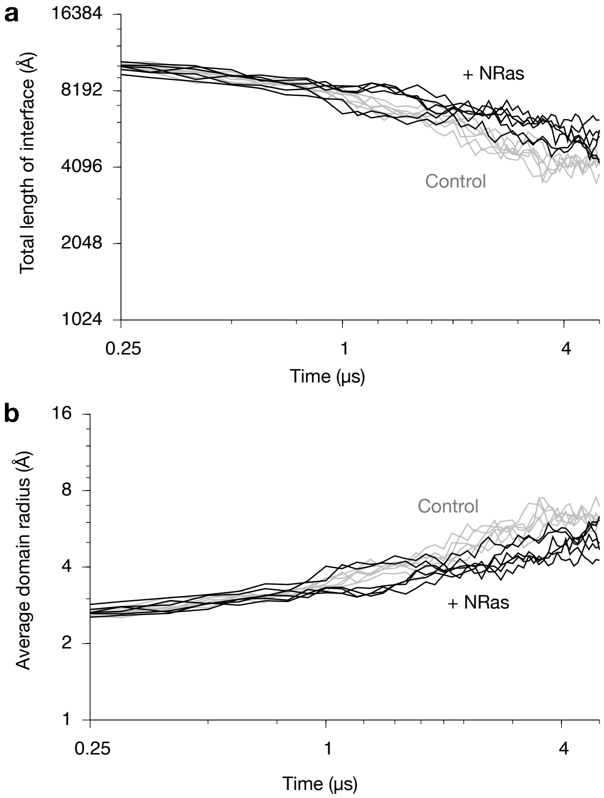

Fig. S8 Log-log plots of how (**a**) the total length of the interface and (**b**) the average domain size vary with time. These suggest that there may be two different regimes, with a boundary at around 2-3 μs.

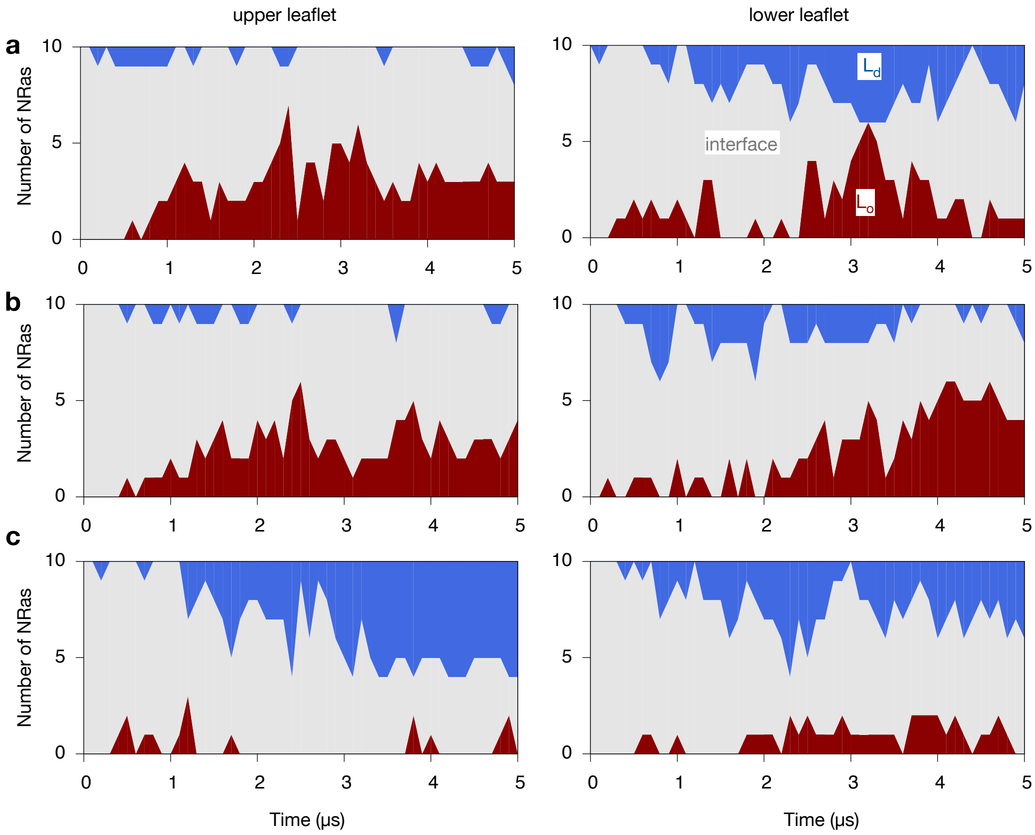

Fig. S9 NRas localises to the interface between the L_o and L_d domains. A protein is defined as belonging to one of the two phases if the 20 Å footprint of the protein contains $> 80\%$ of one phase or the other, otherwise it is categorised as interfacial. The first panel is identical to Fig. 7b in the main body of the manuscript.
